# Diet shifts alter the activity and distribution of digestive enzymes in an herbivorous fish

**DOI:** 10.1007/s10695-025-01567-y

**Published:** 2025-09-18

**Authors:** K. Clerre Rafanan, Michelle J. Herrera, Caitlyn Catabay, Donovan P. German

**Affiliations:** 1 Department of Ecology & Evolutionary Biology, University of California, Irvine, CA 92697-2525 USA; 2 Present Address: Department of Biological Sciences, California State University, Fullerton, CA 92831 USA; 3 Present Address: Marine Biology Research Division, Scripps Institution of Oceanography, University of California, San Diego, San Diego, CA 92093 USA

**Keywords:** Digestion, Herbivory, Aquaculture, Prickleback fish, Physiology

## Abstract

Digestion is primarily performed by digestive enzymes. Here, we examined the activity levels of seven digestive enzymes along the digestive tract of the herbivorous fish, *Cebidichthys violaceus*. We reared *C. violaceus* on carnivore, omnivore, and herbivore diets in the laboratory for nine months and compared the digestive enzyme activities among the fish on the different diets and with wild-caught fish consuming their natural foods. Enzymatic activities were generally lower in the laboratory than in wild-caught fish. The marked anterior-to-posterior amylase activity gradient along the gut in wild-caught fish was absent in the lab-fed fish. We hypothesize that the dampened enzymatic activity may have been caused by reduced food intake in the laboratory in comparison to the wild fish. N-acetyl-β-d-glucosaminidase (NAGase) activity (degrades chitin breakdown products) peaked in the distal intestines of the lab-fed fish, but not the wild fish. The role of this enzyme in the digestive process remains unknown since the lab diets contained no chitin, and its origin may have been microbial. Overall, *C. violaceus* can tolerate diets with a wide range of protein and carbohydrate levels. However, the totality of our data suggests that live algal diets may be best for this herbivorous fish in a captive setting, especially for aquaculture.

## Introduction

Digestion is a chemical process and digestive enzymes are the catalysts (Karasov and Martínez del Rio 2007; Vonk and Western 1984). Fishes possess a full suite of digestive enzyme genes and proteins allowing them to efficiently degrade ingested proteins, lipids, and most carbohydrates (Bakke et al. 2010; German and Herrera 2024; Krogdahl et al. 2024). As with many aspects of the digestive system, digestive enzyme activities show variations with the natural diet of the fish. For example, omnivorous and herbivorous fishes that ingest more algae have elevated amylolytic activities in their guts in comparison to those fish eating a more animal-based diet (German et al. 2016; Jiao et al. 2023; Skea et al. 2005; Zemke-White and Clements 1999). These elevated amylase activities can be maintained, even when the animal is ingesting a low-starch diet in the lab, suggesting that the amylase gene expression (Kim et al. 2014) and enzymatic activity level is matched to starch levels in the natural diet in some fishes (German et al. 2004). The matching of digestive enzyme activity to dietary substrate intake is known as the Adaptive Modulation Hypothesis (AMH; Karasov 1992; Karasov and Martínez del Rio 2007). It has roots in these proportions (Karasov and Hume 1997; Karasov and Douglas 2013; Leigh et al. 2018a):

1 $$\mathrm{Digestibility}\;\propto\;\frac{\mathrm{Digestive}\;\mathrm{enzyme}\;\mathrm{activity}}{\mathrm{Substrate}\;\mathrm{Concentration}}\propto\frac{\mathrm{Gut}\;\mathrm{size}}{\mathrm{Digesta}\;\mathrm{Transit}\;\mathrm{Rate}}\propto\mathrm{Time}$$

The “digestive enzyme activity/substrate concentration” part of Eq. 1 predicts that animals need elevated enzyme activities for a substrate in high concentration if the animal is to achieve high digestibility for that substrate. The “gut size/digesta transit rate” part of Eq. 1 predicts that as the digesta transit rate increases, the size of the gut must also increase to maintain the digestive efficiency (Sibly 1981; Horn and Messer 1992). The equalizer is “time,” since more time would thus allow for lower ratios if time was long enough (Silva et al. 2025). Intake is the main determinant of how fast material moves through the gut, thereby affecting the time variable (more intake means more rapid transit of material, and less time; Sibly 1981; Raubenheimer and Simpson 1998; Karasov and Martínez del Rio 2007). Beyond the support for the AMH for carbohydrase activities, there is some evidence that proteolytic enzymes also match with dietary intake, with more elevated activities in animals that naturally consume more protein (i.e., carnivores), although the pattern is not as stark since all animals need to digest and absorb dietary protein on some level (Chakrabarti et al. 1995; German et al. 2004, 2015; Hidalgo et al. 1999; Jiao et al. 2023).

Because digestive enzyme activities correlate with digestibility (see Eq. 1), enzyme activities have long been used as a metric of digestive performance in wild and captive animal settings (Chakrabarti et al. 1995; Hidalgo et al. 1999; Karasov and Douglas 2013; Karasov and Hume 1997; Lindner et al. 1995). This is particularly true in aquaculture studies (e.g., Furné et al. 2005; Kuz’mina 1996; Li et al. 2006; Xie et al. 2018) and when potential new aquaculture targets are brought into a long-term captive setting. Generally, studies on digestive enzyme activities in wild-caught fishes brought into the laboratory have focused on short-term plasticity in response to dietary shifts (Barman et al. 2005; Leigh et al. 2018b; Xie et al. 2018; Nguyen-Phuc et al. 2021). However, when a fish species is first brought into a long-term (>3 months) culturing environment, what is the impact on their digestive enzyme activity levels? For instance, some investigations revealed decreases in enzymatic activities when fish are brought into captivity (Yang et al. 2018; Djokic 2024; Nguyen-Phuc et al. 2021), whereas others notice no change, or even increases in digestive enzyme activities (German et al. 2010; Han et al. 2020). Many of these changes (or lack thereof) are independent of dietary substrate concentration, suggesting that a captive environment itself can impact digestive physiology (German et al. 2010; Herrera et al. 2025; Turko et al. 2023).

**Fig. 1:**
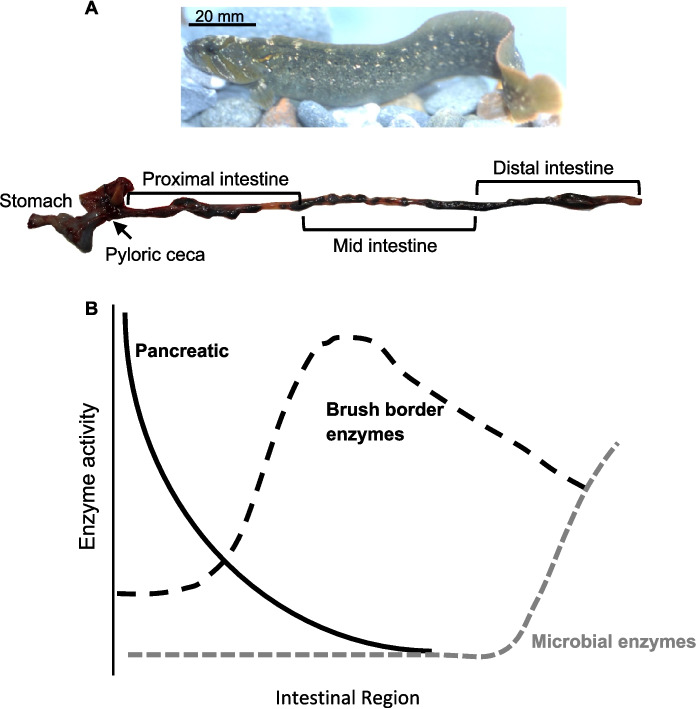
**A***Cebidichthys violaceus* and its digestive system showing the different parts of the gut. For this study, we measured digestive enzyme activities in the pyloric ceca, proximal, mid, and distal intestines of wild-caught fish, as well as those fed carnivore, omnivore, and herbivore diets in the laboratory. Photo by Michael H. Horn. Image modified from Herrera et al. (2025). **B** Expected patterns for pancreatic, brush border, and microbially derived digestive enzyme activities in the *C. violaceus* gut (Wehrle et al. 2020)

As the world grapples with shifting resource availability, it has become clear that fish aquaculture with less reliance on fish meal as a dietary requirement is important for sustainable aquaculture practices (Jobling 2016; Khadim et al. 2025; Lam 1974; Lozano-Muñoz et al. 2022; Merlo et al. 2024; Visca et al. 2017; Xie et al. 2018). Thus, the exploration of herbivorous fishes as aquaculture targets is expanding since such fishes do not need fishmeal in their food and can subsist on plant-based feeds (Lam 1974; Visca et al. 2017; Merlo et al. 2024). One such potential target is the herbivorous prickleback fish, *Cebidichthys violaceus*. Wild-caught individuals of this fish species have been studied across several decades and it is clear that they do indeed thrive on 100% algal diets (Fris and Horn 1993; Horn et al. 1986), have a gut that is optimized for algal digestion (German et al. 2004, 2015), and have some involvement of hindgut bacteria in the digestive process (German et al. 2015; Heras et al. 2020; Herrera et al. 2025). The fish have elevated amylase activities in their guts, underlain by increased copy number of amylase genes in their genome relative to closely related fishes in the same family (German et al. 2016; Heras et al. 2020; Le et al. 2023). *C. violaceus* also has the relatively common pattern of enzymatic activities along their alimentary canals seen in herbivorous fishes, with elevated pancreatic enzyme activities (e.g., amylase and trypsin) in the proximal intestine, elevated brushborder enzymatic activities (e.g., maltase and aminopeptidase) in its mid-intestine, and elevated microbial enzyme activities (e.g., β-glucosidase) in its distal intestine (Fig. 1) (German et al. 2015; Liu et al. 2008; Tengjaroenkul et al. 2000; Pérez-Jiménez et al. 2009).

Given the relative uniqueness of *C. violaceus* as an herbivorous fish that could potentially be cultured for human consumption, we examined how long-term (nine months) exposure to different diets in a laboratory setting impacted digestive enzyme activities in this fish species. We focused on the ability of the fish to digest carbohydrates (amylase, maltase, and N-acetyl-β-d-glucosaminidase), protein (pepsin, trypsin, and alanine aminopeptidase), and lipid (carboxyl ester lipase). We developed three diets meant to resemble herbivorous (~23% protein), omnivorous (~45% protein), and carnivorous (~69% protein) diets (Herrera et al. 2025). We examined how digestive enzyme activity levels changed in the laboratory in reference to wild-caught fish, and how the patterns of activity varied along the gut of the fish.

We hypothesized that the fish would show some plasticity in digestive enzyme activities, in accordance with the AMH (Table 1) (Karasov 1992; Karasov and Martínez del Rio 2007). The AMH has been widely supported for carbohydrase activities appropriate for the natural diet of a given animal. Results have been less clear for proteases, and lipases, and it is also unclear what happens to such enzymatic activities in response to long-term dietary changes (German et al. 2010; Leigh et al. 2022). In this study, we examined what happens to digestive enzyme activities when we first force an herbivorous fish into an artificial setting for a prolonged time period and thus, provides insight into that early period of culturing an herbivorous fish species that has potential to help achieve sustainable aquaculture needs. As a metric of gut size, we measured relative stomach mass (RSM = stomach mass (g) × body mass (g)^−1^) in the fish fed the different diets, predicting larger stomachs in those fish consuming more algae since they would eat more food on a daily basis, consistent with Eq. 1 (Table 1).

**Table 1:** Predicted patterns of stomach size and digestive enzyme activity levels in the intestines of *C. violaceus* fed different diets in the laboratory or captured from the wild

	Diet							
Variable	Lab Carnivore		Lab Omnivore		Lab Herbivore		Wild	
	Peak region	Activity	Peak region	Activity	Peak region	Activity	Peak region	Activity
*Carbohydrases*								
α-Amylase	Proximal	Low	Proximal	Moderate	Proximal	High	Proximal	High
Maltase	Mid	Low	Mid	Moderate	Mid	High	Mid	High
NAGase	Distal	Low	Distal	Low	Distal	Low	Distal	Moderate
*Proteases*								
Trypsin	Proximal	High	Proximal	Moderate	Proximal	Low	Proximal	Low
Aminopeptidase	Mid	High	Mid	Moderate	Mid	Low	Mid	Low
Lipase	Proximal	Moderate	Proximal	Moderate	Proximal	High	Proximal	High
*Stomach size*		size		size		size		size
Relative Stomach Mass	N/A	Small	N/A	Moderate	N/A	Large	N/A	Large

Peak region refers to which region of the intestine (Fig. 1) will have the highest activity for that enzyme and diet combination

NAGase: N-acetyl-β-d-glucosaminidase

Relative Stomach Mass = stomach mass (g) body mass (g)^-1^

## Materials and methods

### Fish capture and feeding trial

Twenty-seven individuals of *Cebidichthys violaceus* were collected by Hand and dipnet in September 2016 at low tide from the rocky intertidal habitat on the central California coast near Piedras Blancas (35.65°N, 121.24°W). Six of the fish were euthanized and dissected in the field to act as representatives of the wild condition for the stomach (these fish were also used in hindgut microbiome analyses in Herrera et al. 2025). All wild-caught fish had full guts, which was desired in this study of an active digestive system (German et al. 2015). The remaining 21 fish were transported to the laboratory at University of California, Irvine (Herrera et al. 2025). At UC Irvine, the fish were transferred to a system of four 76-L cubicle plexiglass aquaria (six cubicles per aquarium, at ~ 13 L per cubicle with the only water exchange among cubicles occurring at the top) connected to a common recirculating system, including a sump, biological, particulate, activated carbon, UV filtration, protein skimmer, and chiller. Each fish was assigned to their own cubicle, which included a 12-cm section of 2.54-cm diameter pvc pipe in which the fish could hide (German et al. 2004; Herrera et al. 2022). The system contained filtered seawater pumped from Newport Bay, CA, and fish were under a 12L:12D Light cycle. The water temperature was maintained at 15 °C (the upper end of temperatures measured at the collection site) with a coil chiller (Aqualogic, San Diego, CA, USA) for the duration of the experiment, and the temperature and chemical conditions (pH and ammonia concentrations) of the tank system were monitored daily to confirm that they did not vary during the experimental period. See Herrera et al. (2025) for more details on the aquarium system and fish diets since this current study used the same fish specimens as Herrera et al. (2025) but focused on enzymatic activities along the whole gut, as opposed to the microbiome and enzymes in the distal intestine only in the former paper. Thus, this current investigation is a complementary study to Herrera et al. (2025).

The fish were randomly assigned to one of three diets, a Lab-Herbivore (LH) diet, a Lab-Omnivore Diet (LO), or a Lab-Carnivore (LC) diet. The diets were not isocaloric, as they were meant to represent a range of possible diets the fish could encounter in the wild or under aquaculture conditions. The LC diet was fish-based (68.8% protein and 14.31 kJ/g), the LO diet was mixed fish-based and algal-based (45.4% protein and 11.04 kJ/g), and the LH diet was algal-based (22.8% protein and 6.06 kJ/g) (Herrera et al. 2025). Fish were fed the diets to satiation two–three times daily for three months to allow them to acclimate to the system. At the fourth month, each fish was anesthetized (0.1 g L^−1^ MS-222), measured and weighed, and returned to their tanks. The fish were fed in this manner for another six months.

At the conclusion of the experiment, fish were fed their morning meal (approximately 0900 h), and within three hours, each fish was euthanized with an overdose of MS-222 (1 g L^−1^ seawater), measured (SL ± 0.5 mm), weighed (body mass, BM ± 0.1 g), and dissected on a sterilized cutting board kept on ice (4 °C). Each digestive system was removed by cutting just anterior to the stomach and at the anus (Fig. 1). The guts were gently uncoiled, measured (gut length, GL). The stomachs, pyloric ceca and livers were excised with a razor blade and placed in their own centrifuge vials, whereas the intestines were divided into three sections of equal length, designated as the proximal, middle, or distal intestine (Fig. 1) (German et al. 2015). Each section was emptied of their contents by pushing with the blunt side of a razorblade and the tissues were frozen separately in individual centrifuge vials in liquid nitrogen. Frozen tissue samples were stored at − 80 °C until analyzed. The relative stomach mass [RSM = stomach mass (g) × body mass (g)^−1^] was calculated. The same procedure was followed for fish dissected in the field.

Gut tissues from individual fish were weighed and homogenized following Rankins et al. (2023). Intestinal tissues were homogenized in 25 mM Tris–HCl (pH 7.5) (German et al. 2015), whereas stomach tissue was homogenized in 100 mM citric acid-sodium citrate buffer (pH 5.0), which better stabilizes the stomach homogenates than pH 2.0 (Rankins et al. 2023). After centrifugation at 9400 × *g* for two minutes at 4 °C, the supernatants of homogenates were collected and stored in small aliquots (100–200 ml) at − 80 °C until just before use in spectrophotometric or fluorometric assays of digestive enzyme activities.

### Assays of digestive enzyme activity

All assays were carried out at 15 °C in duplicate or triplicate using a BioTek Synergy H1 Hybrid spectrophotometer/fluorometer equipped with a monochromator (BioTek, Winooski, VT). All assay protocols generally followed methods detailed in German et al. (2004) and German and Bittong (2009). All reactions were run at saturating substrate concentrations (German et al. 2004, 2015). Each enzyme activity was measured in each gut region of each individual fish, and blanks consisting of substrate only and homogenate only (in buffer) were conducted simultaneously to account for endogenous substrate and/or product in the tissue homogenates and substrate solutions. Activities are reported as μmol product produced·min^−1^·g wet weight of tissue^−1^, except for NAGase, which is reported in nmol instead of μmol.

Pepsin activities were measured in stomach homogenates according to Anson (1938). Briefly, 100 μL of 2% hemoglobin in 60 mM HCl (pH 2) was incubated with 25 μL of homogenate in a microcentrifuge tube for 30 min. The reaction was stopped by adding 200 μL of 5% trichloroacetic acid, and the reaction mixture was centrifuged at 4200 × *g* at 4 °C for 6 min. One hundred microliters of the supernatant was transferred into wells of Greiner UV-star microplates (in triplicate wells), and absorbance was read at 280 nm. Pepsin activity was expressed in U (μmol of l-tyrosine liberated per minute) per gram wet weight of gut tissue based on a l-tyrosine standard curve.

α-Amylase activity was measured using 1% potato starch dissolved in 25 mM Tris–HCl containing 1 mM CaCl_2_. Briefly, five μL of intestinal homogenate was combined with 95 μL of the substrate in centrifuge vials and incubated for 30 min. Reducing sugars released were measured using the Somogyi-Nelson reagents at a wavelength of 650 nm (Nelson 1944; Somogyi 1952; German et al. 2004). The α-amylase activity was determined from a glucose standard curve and expressed in U (μmol glucose liberated per minute) per gram wet weight of gut tissue.

Maltase activities were measured following the glucose oxidase–peroxidase method of Dahlqvist (1968), using o-dianisodine as a dye at a wavelength of 540 nm, as described by German and Bittong (2009). We used 112 mM maltose dissolved in 25 mM tris buffer, pH 7.5. Five microliters of intestinal homogenate was combined with 15 μL of substrate in a centrifuge vial and incubated for 30 min. The maltase activity was determined from a glucose standard curve and expressed in U (μmol glucose liberated per minute) per gram wet weight of gut tissue.

N-acetyl-β-d-glucosaminidase (NAG) activities were measured following German et al. (2011), using a 200 μM solution of 4-methylumbelliferyl-N-acetyl-β-d-glucosaminide dissolved in 25 mM Tris–HCl (pH 7.5). Briefly, 90 μL of substrate was combined with 10 μL of intestinal homogenate in a black microplate and incubated for 30 min. Following incubation, 2.5 μL of 1 M NaOH was added to each microplate well, and the fluorescence read immediately at 365 nm excitation and 450 nm emission. Each plate included a standard curve of the product (4-methylumbelliferone), substrate controls, and homogenate controls, and enzymatic activity (nmol product released per minute per gram wet weight tissue) was calculated from the MUB standard curve.

Trypsin activity was assayed using a modified version of the method designed by Erlanger et al. (1961). The substrate, 2 mM Nα-benzoyl-l-arginine-p-nitroanilide hydrochloride (BAPNA), was dissolved in 25 mM Tris–HCl buffer (pH 7.5) by heating to 90 °C, then chilling to 15 °C for the assay. Ten microliters of intestinal homogenate was combined with 90 μL of substrate, incubated for 30 min, and read at 410 nm. Trypsin activity was determined with a p-nitroaniline standard curve and expressed in U (μmol p-nitroaniline liberated per minute) per gram wet weight of gut tissue.

Aminopeptidase activity was measured using 2.04 mM l-alanine-p-nitroanilide HCl (Roncari and Zuber 1969) dissolved in 25 mM tris buffer (pH 7.5). Five microliters of intestinal homogenate was combined with 95 μL of substrate, incubated for 30 min, and read at 410 nm. Aminopeptidase activity was determined with a p-nitroaniline standard curve, and activity was expressed in U (μmol p-nitroaniline liberated per minute) per gram wet weight of gut tissue.

Lipase (nonspecific bile-salt activated) activity was assayed using 0.55 mM p-nitrophenyl myristate (in ethanol; Iijima et al. 1998) in the presence of 5.2 mM sodium cholate dissolved in 25 mM Tris–HCl (pH 7.5). Ten microliters of intestinal homogenate was combined with 90 μL of bile-salt/substrate mixture, incubated for 1 h, and read at 405 nm. Lipase activity was determined with a p-nitrophenol standard curve and expressed in U (μmol p-nitrophenol liberated per minute) per gram wet weight of gut tissue.

### Statistical analyses

Prior to all significance tests, Levene’s and Bartlett’s tests for equal variances were performed to ensure the appropriateness of the data for parametric analyses, and any datasets that did not meet the assumptions of ANOVA (including homoscedasticity) were transformed using a Box Cox Transformation. All tests were run using R (version 4.0.1). The activity level of each enzyme was compared among the *C. violaceus* fed the different diets and with wild-caught fish for each tissue with ANOVA followed by a Tukey’s HSD with a family error rate of *P* = 0.05. Similarly, within fish fed the same diet (and in wild-caught fish), the activity levels of each enzyme were compared among gut regions with the same ANOVA conditions. Relative stomach mass was compared among the fish on the different diets and the wild-caught fish with ANCOVA (with body mass as a covariate). Note that the intestinal enzyme data on wild-caught fish are from German et al. (2015), whereas all stomach data (mass and pepsin) are from the wild-caught fish from this study. The distal intestine enzyme data for the lab-fed fish were previously published in Herrera et al. (2025) and are included here for comparative purposes with the rest of the gut. Methods among all studies are identical.

## Results

**Fig. 2:**
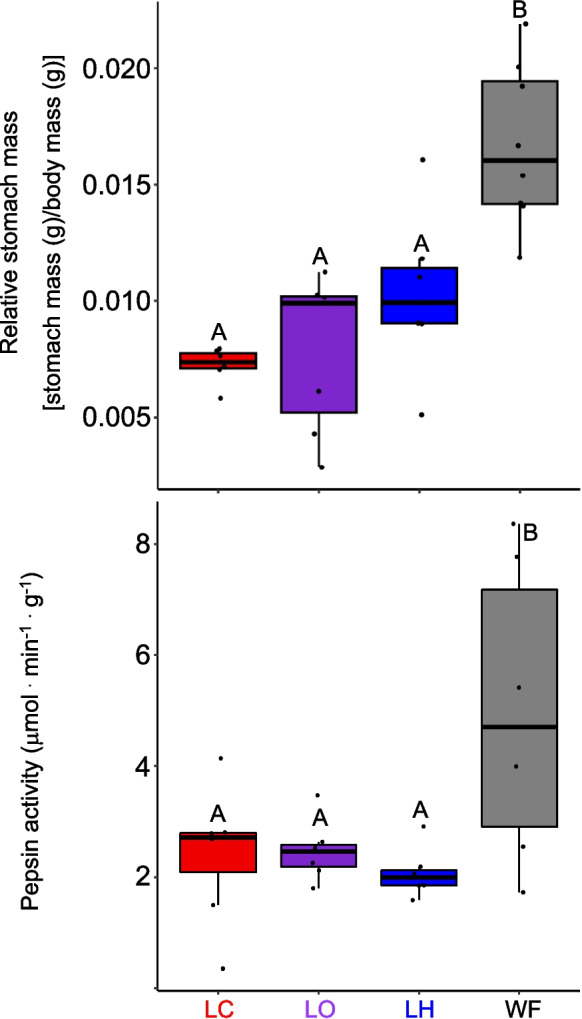
Box and whisker plots of relative stomach mass (top), and pepsin activities (bottom) in the stomachs of *C. violaceus* from the wild (WF), or after consuming different diets in the laboratory for six months. LC = lab carnivore diet, LO = lab omnivore diet, and LH = lab herbivore diet. Relative stomach mass was compared among the fish on the different diets and the wild-caught fish with ANCOVA (with body mass as a covariate), whereas pepsin activities were compared among the fish on the different diets with ANOVA. Both were followed by Tukey’s Honest Significant Difference with a family error rate of *P* = 0.05. Relative stomach mass (diet: *F*_3,23_ = 20.01, *P* < 0.001; body mass: *F*_1,22_ = 7.09, *P* = 0.014), and pepsin activities (*F*_3,23_ = 4.00, *P* = 0.002) showed differences among the fish fed the different diets. Those values sharing a letter on a particular graph are not significantly different

The relative stomach masses (RSM) of the fish varied based on diet, with wild-caught fish having significantly larger RSM than any of the lab-fed fish, which didn’t differ from one another (*F*_3,23_ = 20.01, *P* < 0.001; body mass: *F*_1,22_ = 7.09, *P* = 0.014; Fig. 2). Body mass was a significant co-variate, however. Similarly, pepsin activities were significantly higher in wild-caught fish than the lab-fed fish, which did not differ from one another (*F*_3,23_ = 4.00, *P* = 0.002; Fig. 2).

**Fig. 3:**
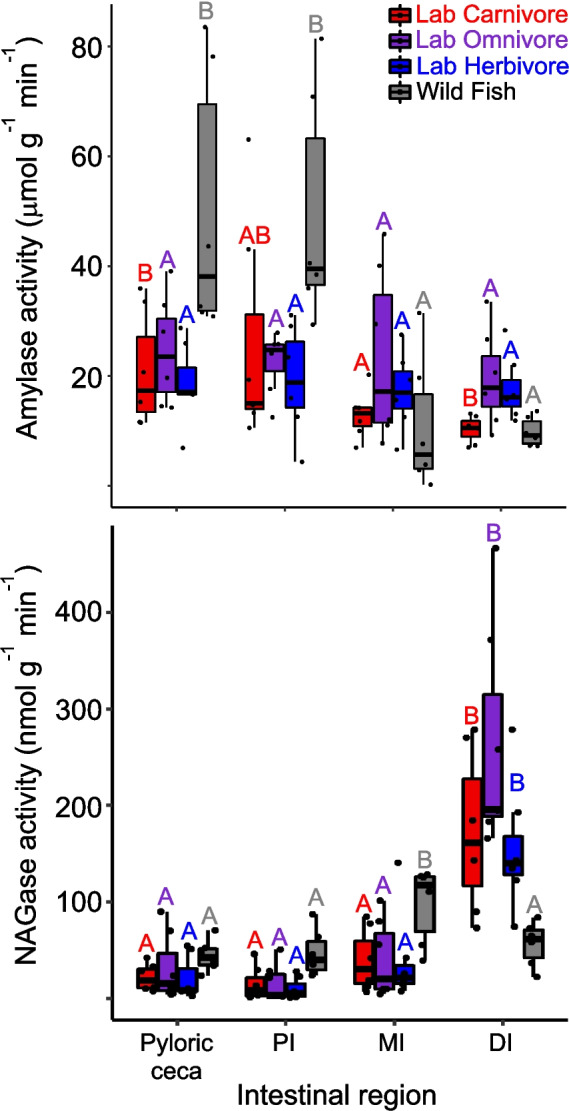
Box and whisker plots of amylase (top) and N-acetyl-β-d-glucosaminidase (NAGase) activities (bottom) in the different regions of the intestines of *C. violaceus* from the wild (WF), or after consuming different diets in the laboratory for six months. Enzyme activities were compared among gut regions for fish on each diet with ANOVA followed by Tukey’s Honest Significant Difference with a family error rate of *P* = 0.05. Statistics for each comparison are in the “Results section” and in Table 2. Activity values for a specific diet and gut region sharing a letter of the same color on a particular graph are not significantly different. Note that the distal intestine enzymatic data for the lab-fed fish are from Herrera et al. (2025) and data for wild-caught fish are from German et al. (2015)

For digestive enzyme activities in the intestine, two patterns emerged that were largely consistent throughout. First, for most enzymes, the wild-caught fish had more elevated enzymatic activity in the relevant gut regions that are expected to show elevated activity for that enzyme (Fig. 1). For amylase, this was the pyloric ceca (*F*_3,23_ = 5.95, *P* = 0.004) and the proximal intestine (*F*_3,23_ = 4.63, *P* = 0.011), where the wild-caught fish had significantly higher activities than the fish on the laboratory diets, which didn’t differ from one another (Fig. 3; Table 2). For maltase, the mid-intestine showed the same pattern (*F*_3,23_ = 4.15, *P* = 0.017; Table 2). For trypsin, it was in the pyloric ceca (*F*_3,23_ = 9.25, *P* < 0.001; Table 2), whereas for aminopeptidase it was the activities of the mid-intestine (*F*_3,23_ = 37.30, *P* < 0.001; Table 2). Lipase was the only enzyme to not show variation among the fish on the different diets. The second pattern was significantly higher activities in the distal intestines of the laboratory-fed fish in comparison to wild-caught fish, which was true for NAGase (*F*_3,23_ = 12.88, *P* < 0.001) and amylase (Table 2; Fig. 3; *F*_3,23_ = 7.38, *P* < 0.001), although for this latter enzyme, it was only the LO and LH fish that were higher than all others.

**Table 2:** Intestinal enzyme activity data in *Cebidichthys violaceus* from the wild, or after consuming different laboratory diets for six months

	Lab carnivore	Lab omnivore	Lab herbivore	Wild	Diet comparison
**Gut region**		Amylase			
Pyloric caeca	^b^20.83 ± 10.05^A^	24.55 ± 9.36^A^	18.43 ± 7.13^A^	^b^50.09 ± 22.23^B^	***F***_**3,23**_** = 5.95** ***P* = 0.004**
Proximal intestine	^b^25.55 ± 19.86^A^	22.60 ± 5.50^A^	19.32 ± 9.39^A^	^b^49.45 ± 19.45^B^	***F***_**3,23**_** = 4.63** ***P* = 0.011**
Middle intestine	^ab^12.93 ± 4.13	23.33 ± 15.21	17.25 ± 6.76	^a^10.95 ± 11.09	*F*_3,23_ = 2.19 *P* = 0.12
Distal intestine	^a^10.29 ± 2.36^A^	19.51 ± 8.40^B^	17.65 ± 5.69^B^	^a^9.82 ± 2.42^A^	***F***_**3,23**_** = 7.38** ***P* = 0.001**
*F*_3,24_	**4.63**	0.31	0.107	**19.78**	
*P*	**0.011**	0.819	0.955	** < 0.001**	
		Maltase			
Pyloric caeca	^a^0.31 ± 0.13	^a^0.38 ± 0.15	^a^0.30 ± 0.06	^a^0.43 ± 0.14	*F*_3,23_ = 1.62 *P* = 0.213
Proximal intestine	^ab^0.62 ± 0.59	^ab^0.75 ± 0.36	^b^0.58 ± 0.16	^b^1.01 ± 0.41	*F*_3,23_ = 1.40 *P* = 0.267
Middle intestine	^b^0.87 ± 0.31^A^	^b^0.96 ± 0.40^AB^	^b^0.77 ± 0.27^A^	^b^1.37 ± 0.27^B^	***F***_**3,23**_** = 4.15** ***P* = 0.017**
Distal intestine	^a^0.45 ± 0.31	^ab^0.68 ± 0.21	^b^0.59 ± 0.30	^a^0.41 ± 0.14	*F*_3,23_ = 1.64 *P* = 0.207
*F*_3,24_	**3.73**	**4.55**	**7.58**	**21.21**	
*P*	**0.025**	**0.012**	** < 0.001**	** < 0.001**	
		N-Acetyl-β-d-glucosaminidase			
Pyloric caeca	^a^21.50 ± 13.11	^a^31.22 ± 34.34	^ab^20.13 ± 22.33	^a^44.23 ± 15.02	*F*_3,23_ = 1.46 *P* = 0.252
Proximal intestine	^a^14.92 ± 16.80^A^	^a^15.86 ± 18.6^A^	^a^9.80 ± 10.88^A^	^a^47.11 ± 22.13^B^	***F***_**3,23**_** = 5.58** ***P* = 0.005**
Middle intestine	^a^38.85 ± 31.17^A^	^a^40.09 ± 38.90^A^	^b^38.34 ± 46.41^A^	^b^97.61 ± 36.28^B^	***F***_**3,23**_** = 3.43** ***P* = 0.034**
Distal intestine	^b^171.53 ± 80.41^B^	^b^262.42 ± 114.30^B^	^c^155.18 ± 64.62^B^	^a^56.32 ± 21.04^A^	***F***_**3,23**_** = 12.88** ***P* < 0.001**
*F*_3,24_	**19.38**	**17.75**	**18.04**	**6.93**	
*P*	** < 0.001**	** < 0.001**	** < 0.001**	**0.002**	
		Trypsin			
Pyloric caeca	0.51 ± 0.43^A^	0.41 ± 0.33^A^	0.65 ± 0.51^A^	^b^1.92 ± 0.84^B^	***F***_**3,23**_** = 9.25** ***P* < 0.001**
Proximal intestine	1.13 ± 1.38	0.98 ± 0.71	0.66 ± 0.44	^ab^1.39 ± 0.45	*F*_3,23_ = 1.07 *P* = 0.380
Middle intestine	1.46 ± 1.50	0.80 ± 0.49	0.63 ± 0.31	^ab^1.13 ± 0.36	*F*_3,23_ = 0.89 *P* = 0.462
Distal intestine	0.45 ± 0.16^A^	0.66 ± 0.34^AB^	0.84 ± 0.32^B^	^a^0.82 ± 0.13^B^	***F***_**3,23**_** = 3.29** ***P* = 0.039**
*F*_3,24_	1.05	1.69	0.39	**6.07**	
*P*	0.39	0.196	0.763	**0.003**	
		Aminopeptidase			
Pyloric caeca	1.05 ± 0.75	^a^1.49 ± 0.89	^a^1.09 ± 0.45	^a^1.73 ± 0.79	*F*_3,23_ = 1.19 *P* = 0.336
Proximal intestine	1.32 ± 0.85	^a^1.67 ± 0.63	^ab^1.74 ± 0.91	^ab^2.29 ± 1.15	*F*_3,23_ = 1.21 *P* = 0.330
Middle intestine	1.61 ± 0.68^A^	^ab^2.01 ± 1.16^A^	^b^2.33 ± 0.91^A^	^c^7.24 ± 1.36^B^	***F***_**3,23**_** = 37.30** ***P* < 0.001**
Distal intestine	2.19 ± 0.43	^b^3.26 ± 1.29	^b^2.51 ± 0.87	^b^3.61 ± 0.70	*F*_3,23_ = 2.87 *P* = 0.059
*F*_3,24_	2.50	**4.29**	**4.36**	**39.96**	
*P*	0.084	**0.015**	**0.014**	** < 0.001**	
		Lipase			
Pyloric caeca	4.19 ± 1.80	^b^3.96 ± 1.74	^b^3.92 ± 2.82	3.98 ± 1.24	*F*_3,23_ = 0.24 *P* = 0.995
Proximal intestine	3.80 ± 1.73	^b^3.91 ± 1.02	^b^3.38 ± 0.87	2.54 ± 1.04	*F*_3,23_ = 1.56 *P* = 0.220
Middle intestine	4.36 ± 2.67	^ab^2.76 ± 1.39	^b^3.75 ± 1.05	2.72 ± 0.89	*F*_3,23_ = 1.71 *P* = 0.192
Distal intestine	3.00 ± 2.48	^a^1.66 ± 1.29	^a^1.07 ± 0.76	2.87 ± 1.45	*F*_3,23_ = 2.19 *P* = 0.116
*F*_3,24_	0.53	**4.34**	**9.38**	2.16	
*P*	0.661	**0.014**	** < 0.001**	0.12	

Values are μmol product produced × min^−1^ × g^−1^, except for N-acetyl-β-d-glucosaminidase, which are in nmol × min^−1^ × g^−1^. Values are mean ± standard deviation. Inter-dietary comparisons within a gut region were made for each enzyme with ANOVA followed by a Tukey’s HSD multiple comparisons test. Capital letters show significant differences across rows, where values not sharing a capital letter are significantly different from one another. Activities were compared across gut regions within fish consuming each diet with ANOVA followed by Tukey’s HSD multiple comparison test. Lowercase letters show significant differences in the columns for each enzyme, where values for a given gut region not sharing a lower-case letter are significantly different. Note that the distal intestine enzymatic data are from Herrera et al. (2025), and wild-caught fish intestinal enzyme data are from German et al. (2015)

In terms of the expected patterns of enzymatic activities moving along the digestive system, the wild-caught fish mostly match the expected patterns shown in Fig. 1 (Table 2). Many of the lab-fed fish vary from these patterns, starting with LO and LH fish for amylase, with no significant differences among gut regions (Table 2). Although most of the fish show the pattern expected for maltase and aminopeptidase, the mid-intestine activities are simply dampened from their peaks in the wild-caught fish. NAGase, again, stands out due to the large distal intestine activity spike seen in the lab-fed fish for this enzyme (Table 2).

## Discussion

The results of this study did not match our predictions that were based on the Adaptive Modulation Hypothesis (Karasov 1992; Karasov and Martínez del Rio 2007). We largely did not observe digestive enzyme activities match with substrate load of the diet. For instance, we predicted that the wild-caught fish and the LH diet fish would have elevated amylase activities in their guts, while the LC diet fish would show the highest trypsin and aminopeptidase activities in theirs (Table 1). Instead, what we generally observed was that the wild-caught fish had more elevated enzymatic activities in the relevant gut region where a given digestive enzyme activity tends to peak. So, wild-caught fish had elevated amylase activity in their proximal intestine, and elevated maltase activity in their mid-intestine, in comparison to the lab-fed fish, regardless of diet, and this was true for nearly every enzyme measured. Thus, what we observed was a general dampening of enzymatic activity in the lab. Moving beyond short-term tests of enzymatic plasticity, we observed something different from the AMH altogether, and instead, are likely seeing differences based on reduced food intake in the laboratory.

One of the most important determinants of gut size is how much an animal eats on a daily basis (Duque-Correa et al. 2024; German et al. 2015; Herrera et al. 2022; Karasov and Martínez del Rio 2007; Leigh et al. 2018a). Intake impacts length and mass of the gut (German and Horn 2006). Relative gut length (Duque-Correa et al. 2024; Kramer and Bryant 1995; Rankins et al. 2023; Ribble and Smith 1983) gets longer in animals that are consuming more food, generally because more intake means more rapid transit of material, and to maintain digestibility, the gut must get longer (Eq. 1), or nutrients would be lost to the feces (Herrera et al. 2022; Karasov and Hume 1997; Leigh et al. 2018a; Raubenheimer and Simpson 1998). However, *C. violaceus* did not show significant variation in relative gut length in this particular feeding trial (Herrera et al. 2025), something that is not uncommon in herbivorous pricklebacks (Herrera et al. 2022), even in long-term feeding trials (German and Horn 2006), since they already have long guts that cannot get much shorter on the scale of months (Herrera et al. 2022; Rankins et al. 2023). For example, the herbivorous prickleback, *Xiphister mucosus*, only shortened its gut by about 13% after consuming a carnivorous diet in the laboratory for six weeks, whereas a carnivorous prickleback, *Anoplarchus purpurescens*, could only lengthen its gut by about 14% on a high-fiber diet over those six weeks (Herrera et al. 2022). Zebrafish, on the other Hand, showed nearly a 30% increase in gut length on a high-fiber diet over months, illustrating that marked plasticity in gut length is possible in some fishes over a long enough time period (Leigh et al. 2018a), but clearly not in *C. violaceus* (German and Horn 2006).

In this study, the lab-fed fish did show smaller relative stomach masses in the laboratory in comparison to wild-caught fish (Fig. 2), similar to what we observed for overall gut mass and distal intestine mass in the same fish, and consistent with Eq. 1 (Herrera et al. 2025). Additionally, *C. violaceus* raised on high-protein diets over months had lighter relative gut masses than wild-caught fish of the same size (German and Horn 2006). Like relative gut length, the relative stomach mass can be a strong indicator of intake, and can get smaller with changing seasons and reduced food availability in wild fish, and in response to lower intake in the laboratory (Gosch et al. 2009; Jobling 1982; Känkänen and Pirhonen 2009; Pirhonen et al. 2019). Thus, it appears that in comparison to wild-caught fish, the lab-fed fish ate less, and thus had smaller stomachs. Wild-caught *C. violaceus* have gut contents that total about 6% of their body mass at any given moment (German et al. 2015), which is likely much higher than what was consumed in the lab on a daily basis.

It is possible that this overall reduced intake is responsible for the reduced digestive enzyme activity observed in the lab-fed fish in this study. In the wild, *C. violaceus* eats algae of relatively lower nutrient content than the diets we fed them in the laboratory (Herrera et al. 2025; Neighbors and Horn 1991). Thus, they have higher intake to meet their nutritional needs (Fris and Horn 1993). The LH diet itself was mostly dried and ground algae (86.5% on a dry mass basis), and although this has a lower protein content than the LO and LC diets, each bite of the LH diet would contain more available nutrients and algal compounds than whole thalate algae eaten by the fish in nature (Fris and Horn 1993; Herrera et al. 2025; Neighbors and Horn 1991). Hence, bite for bite, even the LH diet is fairly nutrient-rich and may have required less consumption on which to thrive in comparison to eating whole algae in nature. In support of this, the fish did not grow significantly differently from one another on the different diets in the laboratory, and this is despite the protein and caloric differences among the diets (see growth data in Herrera et al. 2025).

Lower intake can also help explain the loss of enzymatic activity gradients along the gut. One of the most important determinants of gut transit time is intake: if an animal eats more, digesta traverses the digestive tract faster (Horn and Messer 1992; Karasov and Hume 1997; Raubenheimer and Simpson 1998; Sibly 1981). Faster transit of material requires elevated enzymatic activities to maintain digestibility; otherwise, digesta would be excreted from the gut with relatively low nutrient acquisition (Eq. 1; German et al. 2015; Horn and Messer 1992; Karasov and Hume 1997; Liou et al. 2013; Raubenheimer and Simpson 1998). If intake is lower, as we suppose is occurring in the laboratory, then enzymatic activities can also be lower since enzymes have more time in contact with digesta in any one gut region, but particularly in the region where they are primarily secreted (Karasov and Hume 1997; Leigh et al. 2018a). This means the time variable is larger in Eq. 1. Thus, with lower intake and slower gut transit times, enzymatic activities can be lower in the laboratory than in the wild. The reason we are focused on intake as a potential determinant of enzymatic activity is because nearly all enzyme activities were dampened in the laboratory, regardless of what diet the fish was eating, thus suggesting it was not just changes in nutrient content, as proposed by the AMH.

We hypothesize that pancreatic enzyme activities detected in the distal intestine are a) of pancreatic origin and washed down the gut with digesta (Rothman et al. 2002; Vonk and Western 1984), b) come from diffuse acinar cells in the distal intestine that secrete these enzymes, but at lower levels because there are fewer acinar cells than more proximal intestinal regions, or c) come from microbial sources, especially in a fish like *C. violaceus* with a rich microbial community along their distal intestine mucosa (Herrera et al. 2025; Skea e et al. 2005). It could also be a combination of all three. Pancreatic digestive enzyme gene expression is readily measurable in distal intestine tissue of *C. violaceus* (Herrera et al. 2025), even if it is lower than the expression seen in the proximal regions of the gut (including the pyloric ceca; Heras et al. 2020). However, given that the target substrates for pancreatic enzymes are polymers, and these are enriched in the proximal intestine region of fish (German and Bittong 2009; Skea et al. 2005), this is usually the region where pancreatic enzyme secretion is highest (Rothman et al. 2002), and thus would matter the most in terms of shifting pancreatic digestive enzymatic activities (in this case, a reduction). In an animal like an herbivorous fish with high intake and relatively rapid movement of material through the proximal intestine (Urquhart 1984), pancreatic enzymes would most definitely be carried distally along the gut (Rothman et al. 2002; Vonk and Western 1984). Why pancreatic digestive enzyme activities in the remaining regions of the intestine did not also decrease should be investigated further, but none of the digestive enzymes we measured in this study were differentially expressed genes in transcriptomic analyses of the distal intestine tissues of these same individual fish used in this study, suggesting that gene expression of the enzymes didn’t change in the distal regions of the intestine (Herrera et al. 2025). Perhaps gene expression only changed most in the region with the most acinar cells (i.e., the proximal intestine; Kim et al. 2014; Heras et al. 2020), in which we did not measure gene expression in response to the laboratory diets in *C. violaceus*. We did examine gene expression of the pyloric ceca and mid-intestine in other prickleback fishes consuming different diets in the laboratory over six weeks, and other than trypsin, digestive enzyme genes were not well represented in the differentially expressed genes (Herrera et al. 2022), matching with a general lack of plasticity in digestive enzyme activities in most species, but especially the herbivorous *X. mucosus* (V.I. Peña, K. Steinmann, and D.P. German, unpublished data). A similar argument can be made for aminopeptidase and maltase and their expression in the mid-intestine: the most active regions were the most impacted and exactly why requires more study. Either way, this would affect the pattern of digestive enzyme activities along the intestine, and in this case, eliminated any pattern seen in wild-caught fish. We note that not all fish (e.g., Day et al. 2011; DeGuara et al. 2003; German 2009; Jónás et al. 1983), even in the family Stichaeidae (e.g., the omnivorous *Phytichthys chirus* and the carnivorous *Anoplarchus purpurescens*), show strong patterns of pancreatic enzyme activities along the gut, which further bolsters the view that intake affects these patterns (*P. chirus* and *A. purpurescens* eat less on a daily basis than herbivorous prickleback fishes; German et al. 2015). Thus, herbivorous fish digestion may be more starkly impacted by artificial lab diets than omnivorous or carnivorous fishes.

Despite there being no chitin in any of the laboratory diets, the lab-fed fish had elevated NAGase activity in their distal intestines in comparison to the wild-caught fish. Much like amylase degrades the polymer starch, and then maltase digests the disaccharide maltose, generating glucose for absorption by enterocytes, chitinase degrades the polymer chitin, and NAGase degrades the disaccharide chitobiose, generating the monomer N-acetyl-glucosamine (Jeuniaux 1966). These are endogenous enzymes with genes in the fish genome (Holen et al. 2023; Vervaet 2019). There are dietary differences in chitinase activities among prickleback fishes with different diets: those consuming more crustaceans have elevated chitinase in their stomachs, in support of the AMH (Rankins et al. 2023). Patterns for NAGase have been similar, suggesting that some fishes do target N-acetyl-glucosamine from chitin in their diet (German et al. 2015; Rankins et al. 2023; Vervaet 2019). In some prickleback species (*Phytichthys chirus*), there is elevated NAGase in the distal intestine specifically, suggesting some contribution of microbes to NAGase activities (German et al. 2015; Jhaveri et al. 2015; Leigh et al. 2021). However, the results of this study strongly suggest a microbial source of the NAGase activities in *C. violaceus*, at least on the laboratory diets. A microbial taxon that became common in the laboratory-fed fish (i.e., in the same exact fish specimens as used in this study, and regardless of diet) is in the genus *Paracoccus* (Herrera et al. 2025). Members of this genus can grow on a number of different substrates, including N-acetyl-glucosamine (Gutierrez-Patricio et al. 2021; Liu et al. 2013), and they produce the NAGase enzyme (Xue et al. 2023). We hypothesize that the increased abundance of this microbe led to the elevated NAGase activities in the hindguts of *C. violaceus* in the lab, even though the substrate for that enzyme was not present in the diet (Herrera et al. 2025). Most microbes do not specialize on a single resource (including *Paracoccus*), and an abundance of substrates for one enzyme can lead to increased activities of other enzymes, even if the substrate load does not match (Allison et al. 2014). Hence, we argue that whatever led to an increase in *Paracoccus*, led to more NAGase production, and this should be examined in more detail.

Beyond potential influences of intake and shifting microbiomes on digestive enzyme activity in *C. violaceus*, one piece of data did support the AMH: the distal intestine amylase activity was highest in the LH and LO diet fish and lowest in the LC diet fish (Table 2). Clearly, the LH and LO diets would have more algal starch in them than the completely fish-based LC diet (Herrera et al. 2025). Perhaps some dietary starch escapes to the distal intestine of *C. violaceus* allowing for more microbial contribution to distal intestine amylase on such diets.

In conclusion, we found that bringing *C. violaceus* into the laboratory and feeding them different diets over nine months did not appreciably alter digestive enzyme activities in accordance with the AMH, based on nutrient loads in the diets. Instead, we saw a general dampening of digestive enzyme activities that we hypothesize have more to do with reduced intake in the laboratory than nutrient content, per se. Others have observed similar patterns bringing fish into controlled environments (Djokic 2024; Yang et al. 2018; Nguyen-Phuc et al. 2021). However, the fish grew on all of the diets, especially the LH diet, suggesting that *C. violaceus* can indeed be raised on algal diets in a laboratory setting (Fris and Horn 1993; Herrera et al. 2025). Thus, if *C. violaceus* is to be explored as a potential target for culturing, then identifying appropriate diets that can be made at scale will be an important endeavor. The generally more active gut seen in wild-caught fish could suggest this fish species would thrive in a polyculture environment, where their algal food was grown alongside them, allowing them to feed on live algae instead of dried and ground algae, and this should be explored further.

## Data Availability

No datasets were generated or analysed during the current study.
